# Physical characterization of biphasic bioceramic materials with different granulation sizes and their influence on bone repair and inflammation in rat calvaria

**DOI:** 10.1038/s41598-021-84033-y

**Published:** 2021-02-24

**Authors:** Joviniano Martins de Oliveira Junior, Pedro Giorgetti Montagner, Rafael Coutinho Carrijo, Elizabeth Ferreira Martinez

**Affiliations:** 1 Division of Oral Implantology, São Leopoldo Mandic Research Institute, Campinas, SP 13045-755 Brazil; 2 Division of Cell Biology and Oral Pathology, São Leopoldo Mandic Research Institute, Campinas, SP 13045-755 Brazil

**Keywords:** Biomedical materials, Implants

## Abstract

Biphasic calcium phosphate bioceramics (BCP) consist of a mixture of hydroxyapatite (HA) and beta-tricalcium phosphate (β-TCP) within the same particle. Due to their osteoconductive properties, biocompatibility and resemblance to natural bone, these materials have become a promising and suitable alternative to autologous bone grafting. First, the topography characteristics, specific surface area, and total pore volume of BCP were evaluated using scanning electron microscopy and the BET and BJH methods. Next, this study aimed to evaluate the intensity of the inflammatory process and the bone neoformation capacity of various particle sizes of BCP in the repair of critical defects in the calvaria of rats. A xenogeneic biomaterial was used in the control group. After 30, 60, and 90 days, the animals were euthanized, followed by the processing of the samples to measure the intensity of inflammatory infiltrates and the areas of bone neoformation. Our results indicate that no considerable differences were observed in the inflammatory scores in sites treated with distinct BCP grain sizes. A greater area of bone neoformation was measured in the xenogeneic group at all analysis times, with no substantial differences in bone formation between the BCP particle size in the range of 250–500 µm and 500–1000 µm.

## Introduction

Regenerative procedures performed in areas with bone defects represent a great challenge to dental clinical practice, and the fundamental objective of the various techniques available is to ensure that the repairs of these defects are complete in volume and in bone quality^1,2^. In addition to autogenous bone, several biomaterials have been made available, leading to the neoformation of bone tissues in the repair region^3,4^.

Over the past few decades, considerable effort has been devoted to developing biomaterials for diverse clinical regenerative applications. All of these bone grafts should meet specific requirements in order to develop healthy bone tissue neoformation. Biomaterials must have properties that modulate the severity of inflammatory responses to integrate biomaterials into live tissues^5^. Events such as the migration and formation of blood vessels and the adhesion, migration, differentiation, and proliferation of osteoblasts within a three-dimensionally porous scaffold are directly related to the mechanical, biological, physical and chemical characteristics of biomaterials, such as crystallinity, shape, and dimensions of particles, rugosity, porosity, and interconnectivity of pores^6–8^.

In spite of that, none of the currently available bone grafts possess all the desirable characteristics that such an ideal biomaterial should have. For a better understanding of the progress of the tissue remodeling process, descriptive histological and histomorphometrical analyses are the most used methods for assessing bone repair induced by biomaterials^9^. Physical and chemical properties characterization also play an important role in the biomaterials’ in vivo performance analyses^10^. Thus, the search for near-ideal biomaterials and procedures continues to be a challenge in the field of regenerative procedures.

Calcium phosphate ceramics, mainly hydroxyapatite and β-tricalcium phosphate, whose chemical formulas are Ca_10_(PO_4_)_6_(OH)_2_ and Ca_3_(PO_4)_, respectively, have structural, chemical, and physical similarities with the human bone mineral matrix and their osteoconductive properties are widely known^4,11,12^. These ceramics can be synthesized with dimensions below 100 nm, with a structure similar to the inorganic bone component and the potential to influence the viability and activity of osteoblasts^10,13^.

Biphasic biomaterials, with the presence and homogeneous distribution of hydroxyapatite and β-TCP within the same particle, have an architecture similar to bone tissues, with interconnected microporosity through which cell proliferation permeates^10,14^. While hydroxyapatite particles can remain in implanted sites for many years, β-TCP particles degrade much faster^8,15^. In this context, the search for biomaterials that have bioactivity and degradation patterns that enable bone formation simultaneously has been the objective of several in vivo and in vitro studies, among which biphasic biomaterials have stood out^1,3,16^.

The correlation between the particle size of biomaterials, including those manufactured on a nanoscale, and the properties related to implant biodegradation, triggering response, proliferation, adhesion, and cell differentiation, and the higher speed of bone matrix formation makes further studies necessary. To this end, there is considerable scientific interest in the evaluation of the relationship between the size of various granules and their pores with the aspects related to the intensity of the inflammatory process, the amount of newly formed bone in areas submitted to regenerative procedures, and its biodegradation potential^10,17^.

First, this study sought to describe the morphological characteristics, porosity, and specific surface area of biphasic calcium phosphate bioceramics (HA/β-TCP 60:40 ratio) and a xenogeneic biomaterial. Second, it aimed to evaluate the inflammatory response and compare the bone regeneration of biphasic biomaterials with two different granulations (250–500 µm and 500–1000 µm) associated with poly membranes (lactic acid-glycolic co-acid) with the regeneration of a deproteinized bovine bone mineral biomaterial (250 to 1000 μm) associated with a collagen membrane of porcine origin.

## Results

### Topographic characterization

**Figure 1:**
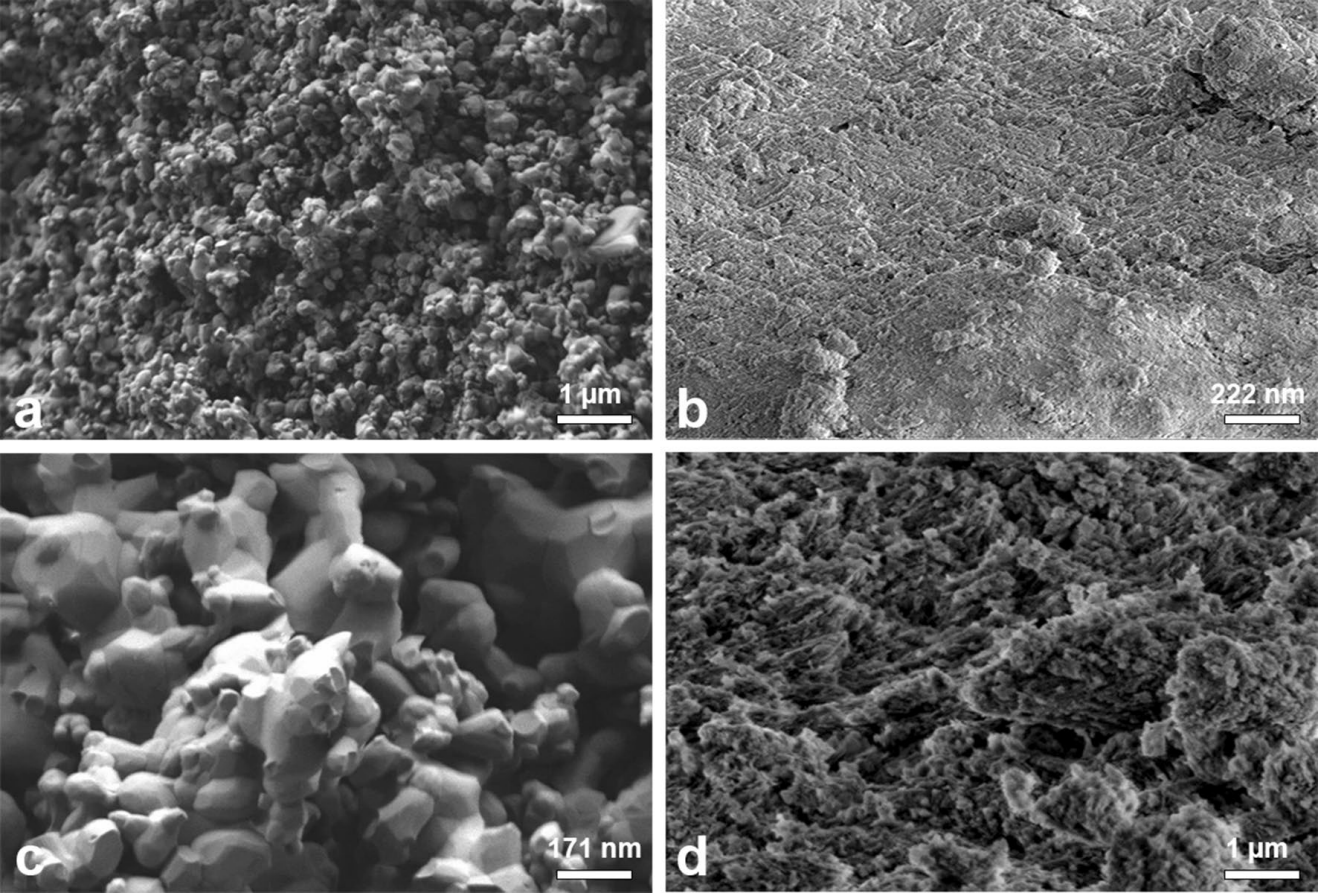
Representative scanning electron microscopy images of the surface structures of the biphasic materials Nanosynt (**a**,**c**) and xenogeneic Bio-Oss (**b**,**d**). Bars: (**a,d**) 1 µm; (**b**) 222 nm; (**c**) 171 nm.

Figure 1 shows the representative scanning electron microscope (SEM) images of the surfaces of the materials. The surface of biphasic materials is composed of interconnected nanometric particles that are interspersed with micro-sized pores (Fig. 1a,c). In the xenogeneic material, a nanoscale surface architecture with greater microporosity and coalescence between the particles was observed (Fig. 1b,d).

Table 1 presents the results of specific surface area (m^2^/g) and the total pore volume (cm^3^/g). Although the biphasic material presented a surface area significantly smaller than the xenogeneic, the total pore volume was higher (p < 0.05).

**Table 1:** Mean (standard deviation), median (minimum and maximum values) of the specific surface area (m^2^/g) and total pore volume (cm^3^/g) of the materials studied (n = 3).

Material	Specific surface area		Total pore volume	
	Mean (standard deviation)	Median (minimum and maximum values)	Mean (standard deviation)	Median (minimum and maximum values)
Nanosynt (G1 and G2)	4.60 (0.71)	4.45 (3.98–5.38)	7.72 (1.47)	7.50 (6.38–9.29)
Bio-Oss (G3)	126.41 (25.47)	115.63 (108.10–155.50)	0.48 (0.08)	0.44 (0.41–0.57)
p-value	p = 0.0143		p = 0.0134	

### Intensity of inflammatory infiltrates

When present, a predominantly lymphocytic mononuclear inflammatory infiltrate was observed in the defect region, permeating grafted biomaterials. Table 2 illustrates the intensity of inflammatory infiltrates in the defect region at different analysis times. In the 30-day time, the score was significantly higher for the biphasic material with higher granulation (G2) when compared with the xenogeneic material (G3) (p < 0.05). However, after 60 days, a decrease in the inflammation score (p < 0.05) emerged. In all the observed periods, the particle size of the biphasic material (G1 and G2) did not influence the inflsammation score (p > 0.05).

**Table 2:** Median (minimum and maximum values) of inflammatory infiltrate scores as a function of materials and time.

Material	Time			p-value
	30 days	60 days	90 days	
	Median (minimum and maximum values)	Median (minimum and maximum values)	Median (minimum and maximum values)	
G1—Nanosynt 200–500 µm	2 (2–3) Aab	2 (2–2) Aa	2 (1–3) Aa	0.7347
G2—Nanosynt 500–1000 µm	3 (3–3) Aa	1 (1–1) Bab	1 (1–1) Bab	0.0015
G3—Bio-Oss 500–1000 µm	0 (0–1) Ab	0 (0–0) Ab	0 (0–1) Ab	0.6432
p-value	0.0033	0.0045	0.0306	

Distinct letters (vertical case and horizontal capital) indicate statistically significant differences (p ≤ 0.05).

Absent (0); Mild (1), up to 25%; Moderate (2), from 25 to 50%; Intense (3), above 50%.

### Histological analysis and bone neoformation within the defect area

**Figure 2:**
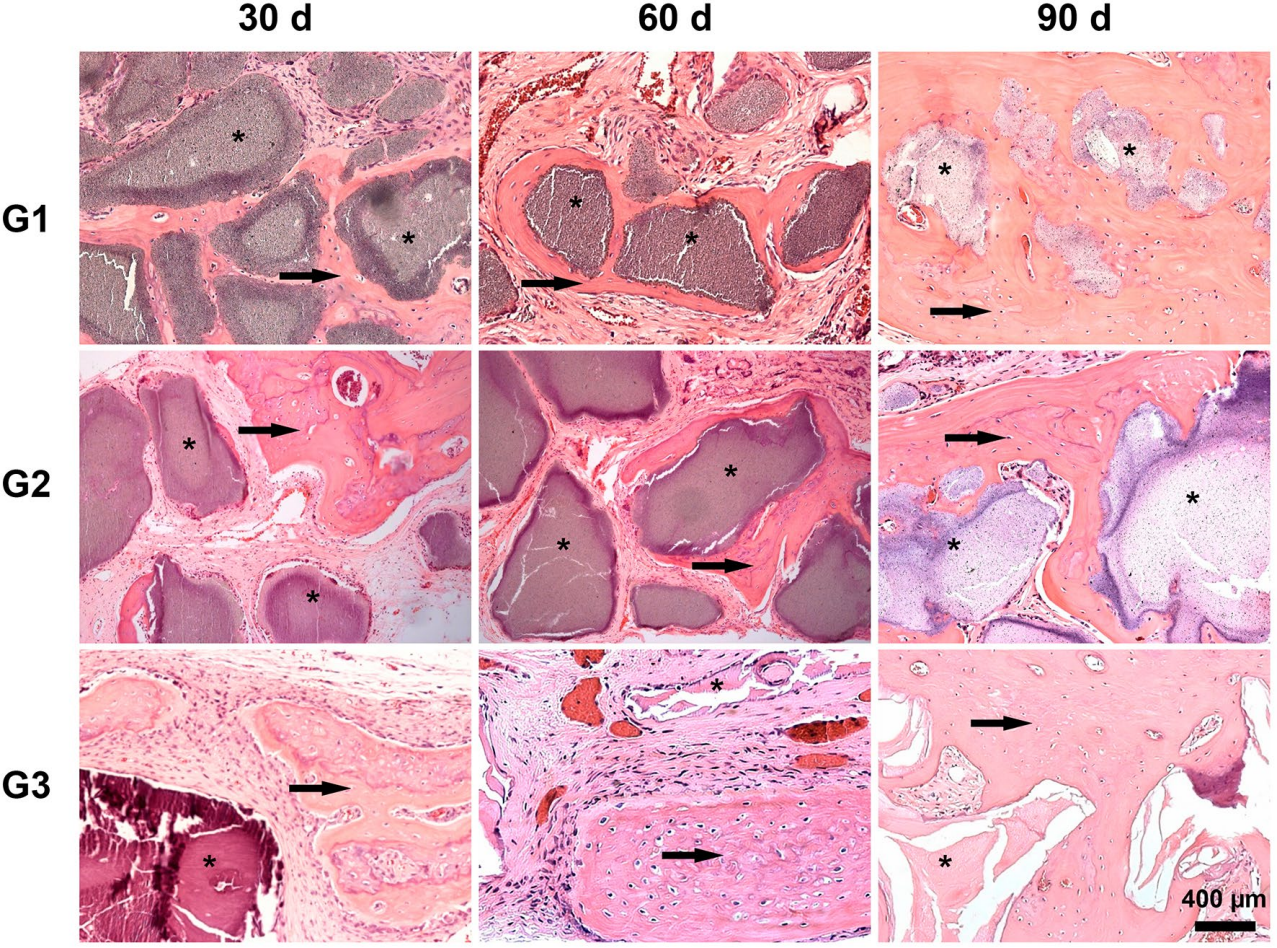
Representative images of histological sections stained with hematoxylin and eosin from the areas of critical-sized bone defects filled with biphasic materials G1 (Nanosynt 250 to 500 μm), G2 (Nanosynt 500 to 1000 μm), and xenogeneic G3 (Bio-Oss 200 to 1000 μm) at 30, 60 and 90 days. At 90 days, a mature lamellar bone was observed, and bone ingrowth occurred both on the biphasic material surfaces and inside its particles. Detailed images showing the presence of remaining biomaterials (*) within the implantation bed, and lamellar bone formation (→). Bar = 400 μm.

Table 3 and Fig. 2 illustrate the results and representative images of bone neoformation in critical defects filled with biomaterials at divergent analysis times. At 30 days, the partial degradation of membranes, collagen fibers arranged in parallel, and congested blood vessels were observed in all specimens. There was no sign of infection in any of the study time points. A diffusely distributed lymphocytic inflammatory infiltrate was mainly observed in G1 and G2, characterizing an inflammatory infiltrate of chronic nature, while in G3 a more discrete process was found. The cellularized and vascularized granulation tissue formed around biomaterial particles was characterized by the presence of vessels and were rich in multinucleated giant cells, especially in both G1 and G2 groups. Additionally, very limited areas of bone neoformation were observed in regions near the stumps and, to a lesser extent, in the center of the defect of all groups. Central regions were characterized by a loose immature connective tissue, and particle surfaces were surrounded by fibroblasts.

**Table 3:** Mean (standard deviation), median (minimum and maximum values) of bone neoformation area (μm^2^) as a function of materials and time.

Material	Time						Multiple comparisons
	30 days		60 days		90 days		
	Mean (standard deviation)	Median (minimum and maximum values)	Mean (standard deviation)	Median (minimum and maximum values)	Mean (standard deviation)	Median (minimum and maximum values)	
G1—Nanosynt 200–500 μm	60,623.29 (24,360.52)	64,376.44 (27,512.79–86,227.48)	115,342.50 (38,054.55)	117,200.10 (70,186.78–156,783.00)	176,245.28 (90,139.08)	183,365.93 (77,098.88–261,150.36)	b
G2—Nanosynt 500–1000 μm	62,709.30 (10,769.56)	58,661.75 (55,198.59–78,315.10)	77,497.73 (43,766.59)	55,632.79 (35,429.82–125,753.14)	112,881.60 (11,727.89)	109,934.97 (102,908.02–125,801.82)	b
G3—Bio-Oss 250–1000 μm	131,158.28 (3064.07)	131,200.28 (128,073.47–134,201.19)	200,789.67 (5083.38)	200,789.67 (197,195.17–204,384.16)	323,712.94 (124,582.58)	323,712.94 (235,619.75–411,806.13)	a
Multiple comparisons	C		B		A		

Distinct letters (vertical case and horizontal capital) indicate statistically significant differences (p ≤ 0.05).

p(material) = 0.0025; p(time) = 0.0004; p(interaction) = 0.7539.

**Figure 3:**
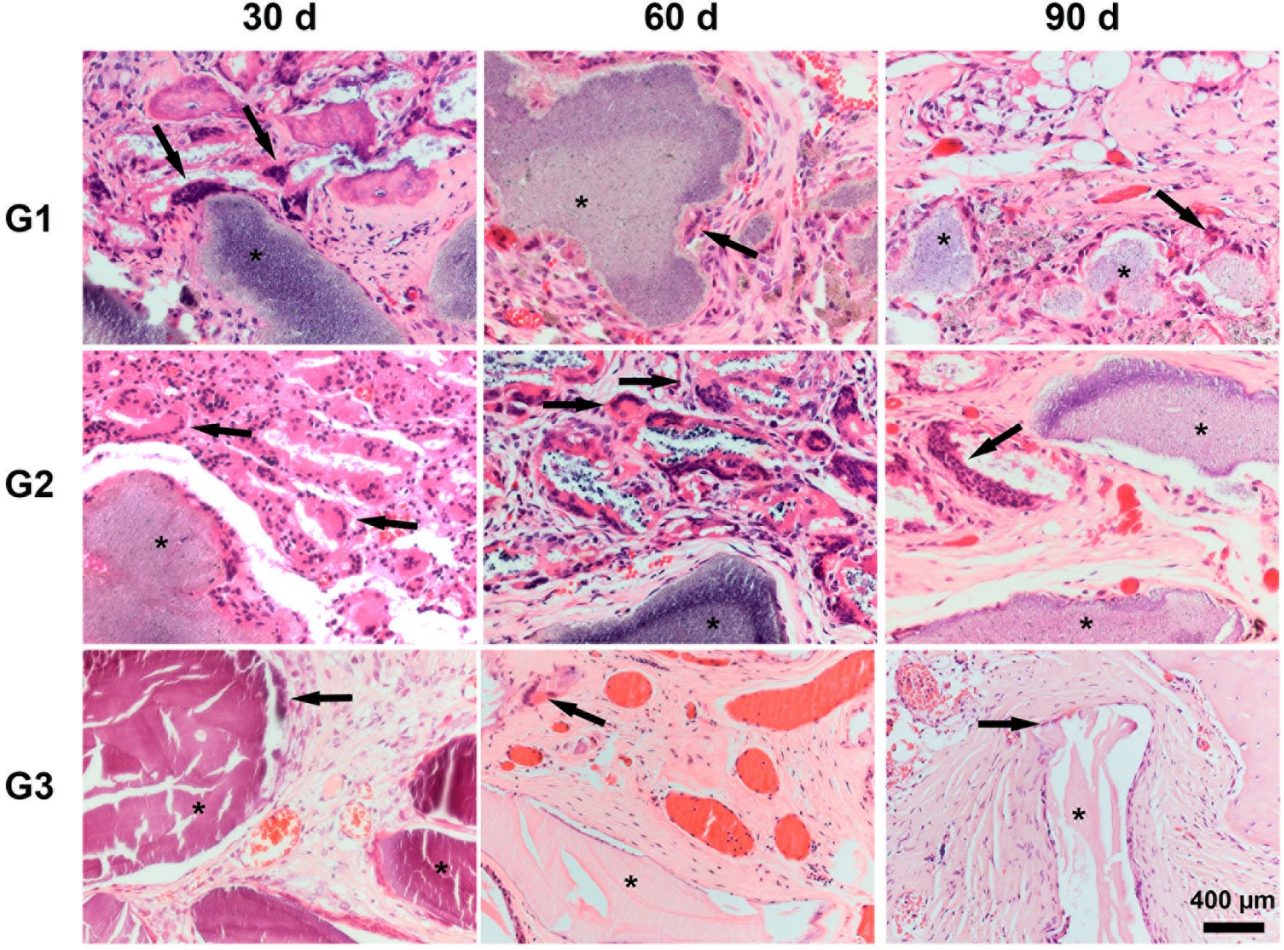
Histological images stained with hematoxylin and eosin from the areas of critical-sized bone defects filled with biphasic materials G1 (Nanosynt 250 to 500 μm), G2 (Nanosynt 500 to 1000 μm), and xenogeneic G3 (Bio-Oss 200 to 1000 μm) at 30, 60 and 90 days. Detailed images showing the presence of remaining biomaterials (*) and foreign body multinucleated giant cells (→) within the implantation bed. Bar = 400 μm.

A slighter inflammatory infiltrate was evident in G1 and G2 at 60 days than at 30 days; however, with the presence of multinucleated giant cells permeated to biomaterials that already had the onset of degradation (Fig. 3). In all groups, membranes were detectable within the implantation bed, showing a stable volume and sustained integrity, and particles were surrounded by mature connective tissue with many well-formed collagen fibers. Inflammatory reaction was characterized by foci of lymphocytes distributed throughout the extent of the G1 and G2 bone defects. In G3, xenogeneic particles remained intact, with no signs of resorption pits on their surfaces, surrounded by loose connective tissue with mild inflammatory infiltrate and multinucleated giant cells around them. Newly blood vessels were distributed among trabecular bones of all specimens, reflecting a high vascularity process in the critical defect. Most of the newly formed bone was limited to the defect margins and directed toward the center portion of it. The formations of newly isolated formed bone were evident in direct contact with particle surfaces in all groups, forming a cohesion unit with the particles, although complete closures of the defects were not observed yet. New bone trabeculae have mature osteocytes in their lacunae. Bone formation exhibited different degrees of maturity ranging from thinner to thicker intercommunicating trabeculations.

At 90 days, in all three groups, there was a reduction in the inflammatory infiltrate, being more relevant than the one found in G3. Multinucleated giant cells were more evident and particle degradation was found in both G1 and G2 groups, whilst few of these giant cells were sporadically found on the surfaces of the xenogeneic particles. Bone formation on the surface and inside of the particles of biphasic material occurred in very close contact, with some particles fully encompassed by mineralized new bone and bone marrow, occasionally yielding complete closure of the defect. Areas close to the defect margin showed a higher degree of direct contact between newly formed bone and xenogeneic particles. In this period, no degradation of the biomaterial was identified for G3; its initial dimensions were well preserved and bone formation occurred in relative proximity to the particles, though only on their surfaces.

Regardless of the time evaluated, G3 presented a larger area of bone neoformation when compared with biphasic materials (G1 and G2, p < 0.05). Moreover, there was no statistically significant difference for biphasic materials with distinct granulations (p > 0.05).

The bone growth area increased significantly with the evaluation time, being higher at 90 days of analysis (p < 0.05).

## Discussion

Regenerative procedures are performed to correct bone defects caused by pathological processes, dentoalveolar trauma, periodontal diseases, alveolar ridge preservation after tooth extraction, and reconstruction of alveolar processes associated with implant treatments^2,4,7^. The combinations of various surgical techniques are necessary, which are simplified by the use of biomaterials, to achieve success in reconstructive procedures^4,18^.

Among the existing possibilities, the autogenous bone graft does not cause immunological responses; it does not offer the risk of the transmission of diseases; and presents osteogenic, osteoinductive, and osteoconductive properties considered the gold standard in reconstructive techniques^8,11,19^. Despite their biological advantages, autogenous grafts also have significant disadvantages, which stimulate the development of new bone substitutes that allow the performance of surgery with lower morbidity and complications^8,10^.

Xenogeneic grafts, especially inorganic bone matrices of bovine origin, have physicochemical characteristics similar to those found in humans^7,20^. The preservation of the volume of the regenerated area is due to the presence of the particles of this biomaterial in the grafted areas for many years^2,18^. This slow resorption can lead to a change in the microarchitecture of the newly formed bone and affect the bone quality obtained, causing it not to meet the requirement of a biomaterial to restore the tissues to their original condition^19,21,22^.

Concerns about the risk of the possible transmission of bovine spongiform encephalopathy and religious issues may impede the use of bovine xenogeneic bone graft materials in some patients^23,24^. To the detriment of its use, the ethical aspects related to the increasing changes in the lifestyle of the segments of society, which act in the defense of animal rights and are contrary to the use and consumption of products of animal origin are also relevant^23^.

Due to the chemical and structural similarities with natural bone apatite, many studies have evaluated the clinical performance of hydroxyapatite, which is one of the bioceramics based on calcium phosphates with better osteoconductive properties, acting as a scaffold for bone growth, besides being biocompatible and bioactive, which is the quality of the graft to be chemically joined to the bone^4,5^. β-TCP is a material that has excellent biocompatibility and, through the action of macrophages and osteoclasts or even because of its own dissolution, ends up presenting rapid resorption in grafted sites^8^. When associated with hydroxyapatite, an implant is formed and denominated as biphasic, and β-TCP is the portion of this ceramic that is absorbed faster^4,8,11^.

In this sense, this study evaluated the osteoconductivity of a low-cost synthetic, which does not offer the risk of the transmission of diseases or cause antigenic reactions. In addition to the origin of the material, physical characteristics related to the form of processing, composition, crystallinity, porosity, shape, and particle size directly influence the speed of the resorption of biomaterials and other aspects related to osteoconduction^6,25^. The particle size is closely related to the microenvironment created by the insertion and compaction of granules inside bone defects and to the three-dimensional architecture obtained in the initial phase of repair^4,12^. Although highly important for clinical practice, the description of each biomaterial’s ideal particle size is not consensual in the literature^7,10^.

In order to develop new biomaterials, it is necessary to understand the issues that surround the integration of the bioceramics with adjacent bone and tissues. For this reason, animal models tests represent a vital step between in vitro tests and human clinical trials, and critical-size rat calvaria model have been successfully used to test both the biosafety and treatment efficacy of novel bone substitute biomaterials^26,27^. The rationale for the selection of this reproducible and cost-effective animal model was based on the fact it has been widely used in several previous studies, and that this kind of cross-sectional investigation permits assessment of bone formation in a reliable manner. Although results obtained from animal model experiments are relevant, extrapolation of findings should not be directly translated to the human situation and results have to be interpreted cautiously.

The analysis of the inflammatory process showed no significant difference in the scores of the groups where biphasic biomaterials were used with distinct granulations so that the particle size did not interfere with the score obtained (Table 2). Although inferring that a milder inflammatory reaction could result in less cellular damage to sites operated with these ceramics and thus contribute to faster bone formation is possible, other studies with larger sample sizes become necessary for their verification.

In all observation periods in G1 and G2, histological findings demonstrated the presence of multinucleated giant cells, similar to the results obtained in another study that has used a biphasic graft with a composition of 60% hydroxyapatite and 40% β-TCP^15^. Conversely, G3, whose biomaterials used have a biological origin and have physicochemical properties similar to those of autogenous grafts^4,20^, presented a much smaller number of these cells in the inflammatory infiltrate (Fig. 3). Therefore, factors such as origin, processing, and physicochemical characteristics of biomaterials cause distinct cellular responses.

The size of the granules was considered one of the characteristics directly involved with the regulation of the degradation of bone substitutes^17^. Thus, biomaterials with lower granulation have a higher speed of disintegration and fragmentation of their particles, with a direct relationship with the earlier recruitment of macrophages, the subsequent fusion of these cells, and an increase in the number of multinucleated giant cells. Although these cells cannot reabsorb particles above 100 μm, the degradation is made by the release of enzymes and the formation of a microenvironment with acid pH, which would lead to the demineralization of adjacent bone and the release of calcium and phosphate ions, resulting in the improvement of local conditions for bone neoformation to occur^4,28^.

In this study, however, multiple comparisons of the results of the areas of bone neoformation showed no significant difference in the bone formation of synthetic biphasic biomaterials with different granulation sizes. In the three times analyzed, the study revealed that there was a significant area of bone growth in G3. In all groups, progressive increases in the area of bone neoformation were observed from the beginning to the end of the experiment. There was no evidence of the encapsulations of the biomaterials used, and the differentiation of osteoprogenitor cells on the ceramic scaffold proved the biocompatibility and osteoconductive effects of the tested materials.

Given that no significant difference existed in bone neoformation when biphasic biomaterials with divergent granulations were used, the option for smaller or larger particles should fall on professional preference, the ease of manipulation and insertion, combined use with other materials, and the clinical condition that requires the regenerative procedure.

The biphasic biomaterials were incorporated into adjacent tissues by processes that involved bone growth around and inside the particles in close contact with surfaces that presented degradations. This condition is considered ideal for an osteoconductive material because the newly formed bone tissues in direct contact with ceramics are formed to replace the biomaterial that is gradually resorbed^29^. This bone formation capacity of bioactive materials forms a strong single interface and from the creeping substitution process, which is the degradation of the resorbable bone substitute accompanied by the simultaneous growth of a new bone tissue^30^. Unlike biphasic materials, the xenogeneic graft showed no signs of degradation, which can be explained by the fact that it is a biomaterial of high crystallinity^12,19,21^.

The main reason for using a biphasic material, which has distinct resorption velocities of its two components, is to enable that as β-TCP degradation occurs, spaces will appear around the granules through which there will be bone growth, while hydroxyapatite will fulfill the function of being an osteoconductive scaffold^30^. Thus, the cells are guided so that there is a bone formation in close contact with the surfaces of the particles, including the formation of bone bridges between the grafted granules^11,15,17^. For biphasic bioceramics, a HA and β-TCP ratio within the range of 65:35 to 55:45 is considered ideal for the resorption of the material simultaneously to the bone formation by replacement^15,31,32^, and the biphasic biomaterials of the test groups have a ratio of 60:40.

The initial resorption of the biphasic biomaterials leads to an increase in the concentration of calcium and phosphate ions, which would be related to increased osteoblastic activities and would inhibit osteoclastic activities. Furthermore, the dissolution of calcium sulfates in implanted sites results in an acceleration of bone regeneration due to the decrease in pH in the region, followed by bone demineralization around it and the consequent release of BMPs^33^.

A higher specific surface area has a direct relationship with the increase in the adsorption of macromolecules and proteins, particularly involved with the induction of osteogenesis^4,8,25,30,34^. In this study, the significant difference in the results of the specific areas of surfaces obtained in the groups evaluated (Table 1) can be explained by the characteristics of the xenogeneic material, which has greater microporosity and is fully processed from bovine medullary inorganic bone, while the test groups are synthetic materials, whose own processes of sintering of its particles result in lower values in this parameter^4,14^.

By contrast, biphasic materials have a significantly higher total pore volume, a property that allows an increase in the permeability of interstitial fluids, the development of a capillary system, and the migration of new bone cells^4,8,26,34^. The diameter of interconnected pores should be at least 100 μm although 300 μm was considered the recommended minimum diameter to allow angiogenesis and bone neoformation inside existing porosities^6^. The biphasic material has a porosity considered dynamic due to the process of the progressive resorption of β-TCP, which is different from what occurs with xenogeneic graft. Accordingly, the progressive increase in the porosity of biphasic material as a function of time may bring some benefits to bone neoformation and angiogenesis^25,34^.

Finally, the present study has some limitations that should be taken into consideration. The first limitation is the small sample sizes. Second, despite having investigated physical characterizations of the biphasic and xenogeneic material, this study is limited by the lack of information on other physical–chemical properties that are very important for the evaluation of biomaterials, including the Fourier transform infrared spectroscopic (FTIR) and X-ray diffraction (XRD) techniques^9,35^. Another limitation was that the aspects related to cytotoxicity, biodegradation, and biocompatibility of these bone substitutes were also not evaluated in this study.

## Conclusion

The results of the present study demonstrate that biphasic ceramics have a higher total pore volume and a smaller specific surface area than xenogeneic materials. For all observed periods, the different particle sizes of the biphasic materials did not influence the inflammation score. This osteoconductive bioceramic was able to promote new bone formation in critical defects created in the calvaria of rats, with no statistically significant differences between the two distinct granulations tested. The micro- and macro-porosity of these particles and the creeping substitution process allowed the occurrence of bone ingrowth, both on their surface and inside them. Despite the smaller amount of bone neoformation, thicker lamellar bone bridging was observed among the synthetic biomaterials. Given that no significant difference existed in bone neoformation when divergent granulations of biphasic calcium phosphate bioceramics were used, these results suggest that the option for smaller or larger particles should fall on professional preference, the ease of manipulation and insertion, and the clinical condition that requires the regenerative procedure.

## Materials and methods

### Morphological characterization and porosities

The analyses of the morphologies of biphasic ceramics and xenogeneic biomaterials were performed by employing scanning electron microscopes with field emission sources (FEG ZEISS Model Auriga and FEI SEM Magellan 400L), respectively, using secondary electrons accelerated at 5 kV under a high vacuum condition with the metallization of samples with a gold conductive film of approximately 20 nm. The micrographs were obtained with increases ranging from 200 × to 40,000 ×.

The Brunauer–Emmett–Teller (BET) and Barrett–Joyner–Halenda (BJH) methods were used to characterize the specific surface area and total pore volume, respectively. The samples were previously heated at 100 °C for 24 h and subsequently immersed in a glass tube containing liquid nitrogen at − 196 °C under vacuum conditions. The data were obtained from the adsorption of high purity gaseous nitrogen in the tested samples, and the specific surface area was determined according to BET theory. Thereafter, on the basis of the adsorption curve of the material, nitrogen desorption in isotherms was performed to determine the average pore volume by the BJH method.

### Animals

For this study, 45 male Wistar rats of the *Rattus norvegicus albinus* were used, with prior approval by the São Leopoldo Mandic Institute and Research Center Ethics Committee on Research for Animal Experimentation (Ethical approval number 2018/20). All surgical procedures were performed in accordance with the directives of this Committee, and the Brazilian regulatory authority for the use of animals for experimental purposes. The authors declare that this study was carried out in compliance with the ARRIVE guidelines. The animals were kept under controlled conditions of temperature and lighting, with a light–dark cycle of 12 h and balanced feeding and water ad libitum.

### Critical size calvarial defect experiment

**Figure 4:**
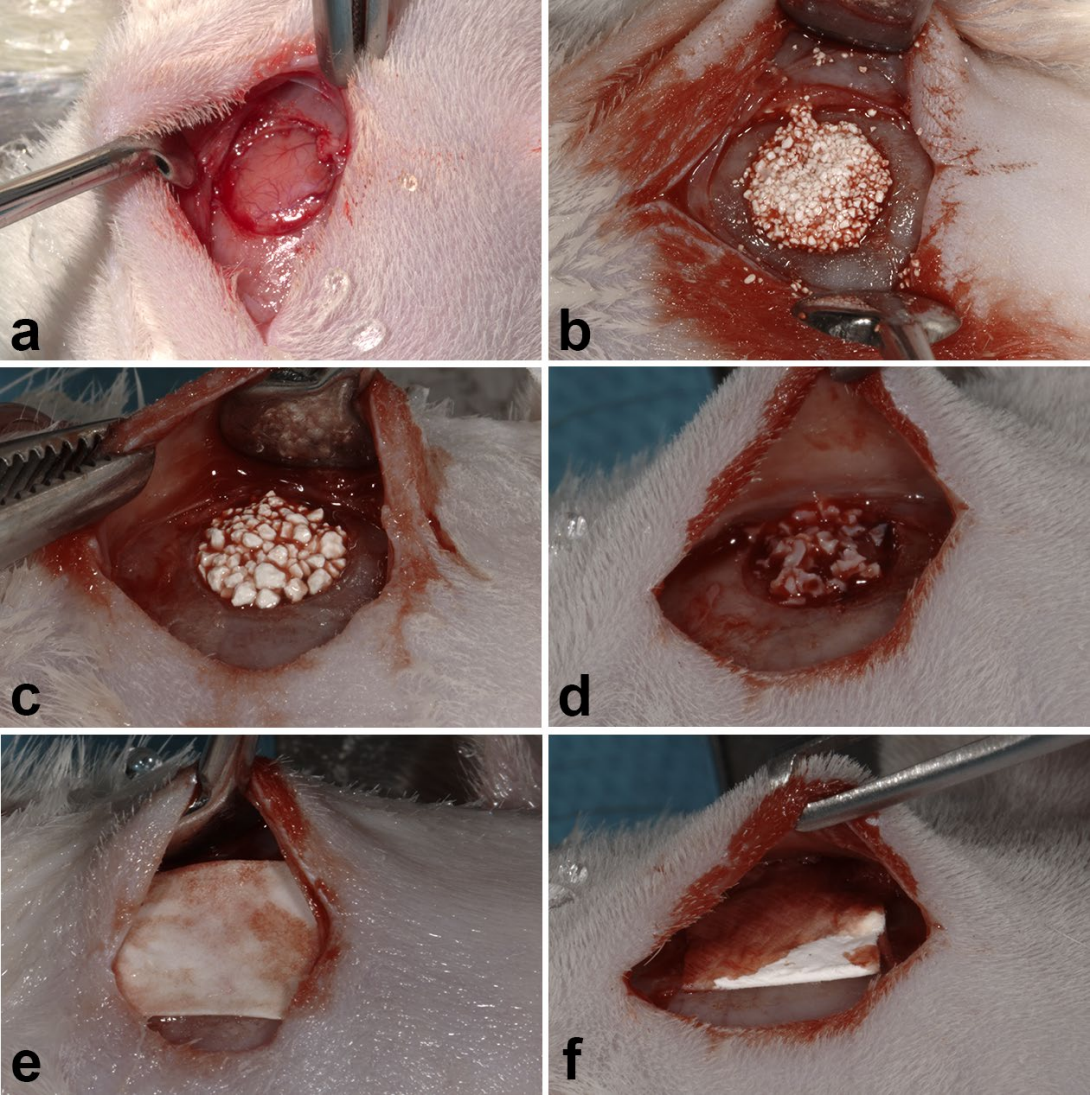
Images of surgical procedures. Dura mater exposure (**a**), defect filling with G1—Nanosynt 250 to 500 μm (**b**), G2—Nanosynt 500 to 1000 μm (**c**), G3—Bio-Oss 250 to 1000 μm (**d**), covered with Duosynt (**e**) and Bio-Gide membrane (**f**).

The animals were divided into three groups according to 30-, 60-, and 90-day evaluation times, totaling 45 evaluation sites. Surgical procedures were performed as previously described. Briefly, animals were anesthetized by an intraperitoneal injection of 5% ketamine hydrochloride and xylazine hydrochloride at 2%. In each animal, the critical bicortical bone defect, involving the external and internal cortical of the calvaria, was created in the parietal bone by means of a 6-mm diameter trephine drill mounted on an implant contra-angle handpiece under abundant external irrigation with a 0.9% saline solution. The internal cortical was removed, releasing it completely from the dura mater and preserving this intact structure (Fig. 4a).

Randomly, the critical defects of the three groups were filled with bone substitutes and resorbable membranes. Two experimental groups were filled with synthetic materials based on biphasic calcium phosphate, 60% hydroxyapatite, and 40% tricalcium β-phosphate (Nanosynt, FGM, Joinville, SC, Brazil) covered by poly membranes (lactic acid-glycolic co-acid) (Duosynt, FGM, Joinville, SC, Brazil). In Group 1 (G1), the particles of the materials had dimensions from 250 to 500 μm and from 500 to 1000 μm in Group 2 (G2). In the positive control group (G3), the xenogeneic material of bovine origin (Bio-Oss, Geistlich Pharma AG, Wolhusen, Switzerland) was used, with particles from 250 to 1000 μm, in combination with a collagen membrane of porcine origin (Bio-Gide, Geistlich Pharma AG, Wolhusen, Switzerland) (Fig. 4).

### Histological processing

Calvarias were demineralized in 20% formic acid, dehydrated and included in histological paraffin, to perform cuts in the central region of the defects, parallel to the median suture, with 4-μm thickness.

The samples were submitted to hematoxylin–eosin staining and later mounted on slides with resin for photomicrographs. The images of the slides were captured in a computerized imaging system (AxioVision rel 4.8, Carl Zeiss) coupled with the light microscope Axioskop 2 Plus (Carl Zeiss, Oberkochen, Germany).

### Histological and histomorphometry analyses

For qualitative and quantitative histological analyses, the osteogenic potential of the materials was considered, describing bone neoformation at the edges of defects and the presence of inflammation, including the presence of blood vessels, giant cells, leukocytes, and phagocytic infiltrates of the evaluated tissues, at different analysis times (30, 60, and 90 days). The classification score was adopted, ranging from 0 to 3, considering the extent of the inflammatory process and predominance of a particular type of inflammatory cell in the defect area, being 0 for absent, 1 for mild (up to 25%), 2 for moderate (from 25 to 50%), and 3 for intense (above 50%)^16^.

Six areas of each slide were randomly chosen, both at the edges of the defect in the calvaria and in its center, and histomorphometric measurements were obtained using the ImageJ image analysis program (National Institute of Health, Maryland, USA). The osteogenic potential of the materials was considered, measuring the areas occupied by newly formed bones in μm^2^.

### Statistical analysis

The t-test was used for heterogeneous variances to compare the data of the surface area and the total pore volume of the materials used. The scores of the inflammatory process were also examined by the Kruskal–Wallis and Dunn tests. The analyses were performed in the R Core Team 2019 program with a significance level of 5%.
